# Establish a New Diagnosis of Sarcopenia Based on Extracted Radiomic Features to Predict Prognosis of Patients With Gastric Cancer

**DOI:** 10.3389/fnut.2022.850929

**Published:** 2022-06-28

**Authors:** Xiao-Dong Chen, Wen-Jing Chen, Ze-Xin Huang, Li-Bin Xu, Hui-Hui Zhang, Ming-Ming Shi, Yi-Qi Cai, Wei-Teng Zhang, Zhao-Shen Li, Xian Shen

**Affiliations:** ^1^Department of Gastrointestinal Surgery, The First Affiliated Hospital, Wenzhou Medical University, Wenzhou, China; ^2^Department of Gastrointestinal Surgery, The Second Affiliated Hospital, Wenzhou Medical University, Wenzhou, China

**Keywords:** radiomics, sarcopenia, gastric cancer, prognosis, diagnosis

## Abstract

**Background:**

Preoperative sarcopenia is a prognostic risk factor for gastric cancer (GC). This study aimed to determine whether radiomic sarcopenia features on computed tomography (CT) could be used to diagnose sarcopenia preoperatively, and whether they could be used to accurately predict the postoperative survival and complication prognosis of patients with GC.

**Methods:**

We retrospectively analyzed data of 550 patients with GC who underwent radical gastrectomy. The patients were divided into training (2014–2016) and validation (2017–2019) cohorts. We established a radiomics-based diagnosis tool for sarcopenia. Thereafter, univariate and multivariate analyses of diagnostic factors were carried out. Receiver operator characteristic (ROC) curves and area under the curve (AUC) were used to compare different diagnostic models. The Kaplan–Meier method was used to estimate the survival curve.

**Results:**

Radiomic sarcopenia correlated with complications and long-term survival. Skeletal muscle index, grip strength, and walking speed were correlated with postoperative complications in both cohorts (AUCs: 0.632, 0.577, and 0.614, respectively in the training cohort; 0.570, 0.605, 0.546, respectively, in the validation cohort), and original sarcopenia was more accurate than any of these indicators. However, radiomic sarcopenia has a higher AUC in predicting short-term complications than original sarcopenia in both groups (AUCs: 0.646 vs. 0.635 in the training cohort; 0.641 vs. 0.625 in the validation cohort). In the training cohort, the overall survival time of patients with original sarcopenia was shorter than normal patients (hazard ratio, HR = 1.741; 95% confidence interval [CI], 1.044–2.903; *p* = 0.031). While radiomic sarcopenia had a greater prognostic significance, the overall survival time of patients with radiomic sarcopenia was significantly worse than normal patients (HR, 1.880; 95% CI, 1.225–2.885, *p* = 0.003).

**Conclusion:**

Extracted sarcopenia features based on CT can predict long-term survival and short-term complications of GC patients after surgery, and its accuracy has been verified by training and validation groups. Compared with original sarcopenia, radiomic sarcopenia can effectively improve the accuracy of survival and complication prediction and also shorten the time and steps of traditional screening, thereby reducing the subjectivity effects of sarcopenia assessment.

## Introduction

Gastric cancer (GC) is the fifth most common cancer, the third leading cause of cancer-related deaths worldwide (1), and the most common malignant tumor in China (2). Radical gastrectomy remains the standard of care for curable GC (3). However, gastrectomy may be accompanied by many complications, such as infection, bleeding, anastomotic fistula, or organ dysfunction (4). Therefore, complications will affect functional recovery and may prolong hospital stays, increase economic burden, and deplete medical resources (5).

It is well known that GC is an extremely aggressive malignant tumor of the upper digestive tract. Owing to gastrointestinal insufficiency or failure, patients with GC often have insufficient nutrient intake or malabsorption. Approximately 60.2% of patients with gastroesophageal tumors develop malnutrition, this percentage is higher than that for most other malignant tumors (6). Therefore, patients with GC often experience malnutrition or cachexia before surgery, increasing the chance of postoperative complications and mortality (7, 8). Therefore, an effective tool for predicting postoperative complications and mortality will be helpful.

Sarcopenia is a malnutrition-related syndrome characterized by the gradual and complete loss of skeletal muscle mass and strength (9) and has been shown to be a new predictor of postoperative complications in patients with GC (10). In 2010, the European Working Group on Sarcopenia in Older People (EWGSOP) reached a general consensus on the definition of sarcopenia: the loss of skeletal muscle plus low muscle strength and/or poor physical function that appears with age (9). Similarly, sarcopenia was described as an old age-related syndrome by the Asian Working Group for Sarcopenia (AWGS); the AWGS also recommended a definition using Asian cutoff values in 2014 (11). However, the traditional method for diagnosing sarcopenia not only needs to consider the waist muscle mass measured on imaging but also muscle strength and physical performance (9). This means that the correct diagnosis of preoperative sarcopenia requires more time and medical resources.

In recent years, as a new method of diagnosis and prediction, radiomic features have been increasingly used for the individualized treatment of tumors (12, 13). Young proposed a new diagnosis method for sarcopenia based on convolutional neural network and radiomics, which proved the feasibility of radiomics in the diagnosis of sarcopenia. However, he did not include muscle strength or physical performance in the sarcopenic auto-diagnosis model, which left their model of sarcopenia somewhat limited (14). In this study, we propose the concept of “radiomic sarcopenia” using sarcopenia features extracted from three-dimensional imaging. Our study aimed to determine whether this new method could be used to diagnose sarcopenia more quickly and objectively before surgery, and whether it could be used to predict postoperative survival and complication prognosis of patients with GC more accurately than the conventional method.

## Materials and Methods

### Inclusion and Exclusion Criteria

We retrospectively recruited patients who were diagnosed with gastric adenocarcinoma by preoperative gastroscopy in two affiliated hospitals of Wenzhou Medical University from December 2014 to June 2019 and who were able to undergo radical surgery (614 patients). The exclusion criteria included patients who refused surgery or were switched to palliative surgery during the operation (20 patients), patients who did not undergo preoperative imaging examination or for whom imaging data were unavailable (35 patients), patients with other tumors or serious organic diseases (6 patients), and patients who were lost to follow-up or for whom the total follow-up time was less than 1 year (3 patients). A total of 550 patients were finally collected and analyzed. All patients underwent radical gastrectomy, and all operations were performed by senior surgeons who independently performed radical gastrectomy for more than 200 cases. The management of GC treatment during the perioperative period was based on the 2010 edition of the Japanese Gastric Cancer Treatment Guidelines (15). This study was approved by the Ethics Committee of the two affiliated hospitals of Wenzhou Medical University and conformed to the tenets of the Declaration of Helsinki.

### Data Collection

The following variables were collected for each patient: (1) clinical characteristics, including age, gender, body mass index (BMI), nutritional risk screening (NRS-2002) score, and the tumor–node–metastasis (TNM) stage of the tumor; (2) operative information, including gastrectomy range, method of reconstruction, laparoscopic surgery and organ combined resection. Postoperative complications were defined as grade II and above surgical outcomes in accordance with the Clavien–Dindo classification (16). Patients were followed up telephonically or with hospital recalls, and survival status and tumor recurrence were recorded. The follow-up frequency was once every 3 months for the first year, once every 6 months for the second to fifth year, and then once a year. The last follow-up date was in February 2021.

### Research Groups

In a chronological order, a time-dependent grouping method was adopted to divide the patients with gastric malignant tumors into either a training cohort (261 cases from 2014 to 2016) and validation cohort (289 cases from 2017 to 2019). Patients in the training group were included in the selection of sarcopenia-related radiomic features. Patients in the validation group did not participate in the screening of features or the establishment of a prognostic model of sarcopenia, they were involved solely in accuracy verification of the model.

### Diagnosis of Original Sarcopenia

The range of skeletal muscles can distinguish from other tissue between –29 and +150 Hounsfield units scale in CT scan (17). Muscle area was calculated using a dedicated processing system (INFINITT PACS software, version 3.0.11.3, BN17 32 bit; INFINITT Healthcare Co., Ltd., Seoul, South Korea). At the cross-section of the third lumbar vertebra (L3), the areas of all skeletal muscles of the patient (the psoas, erector spinae, quadratus lumborum, transversus abdominis, external and internal obliques, and rectus abdominis) were measured preoperatively, and the sum of these areas was calculated as described previously (17). Low muscle mass was defined as L3 skeletal muscle index (SMI) ≤ 40.8 cm^2^/m^2^ for men and 34.9 cm^2^/m^2^ for women (18). To minimize bias, two professionally radiologists completed the muscle area measurement independently and were blinded to patients’ clinicopathological data.

Muscle strength and physical function were determined by measuring preoperative grip strength and 6-m usual gait speed, respectively. Each patient was required to squeeze an electronic hand dynamometer (EH101; Camry, Guangdong Province, China) to obtain the preoperative grip strength. Low muscle strength was defined as a hand grip strength <26 kg for men and <18 kg for women (11). Patients were asked to walk 6 m at their normal speed, and the duration was recorded to calculate the 6-m usual gait speed. Low muscle performance was defined as a 6-m usual gait speed <0.8 m/s (11).

Based on the EWGSOP and AWGS (11), patients with low skeletal muscle mass plus low muscle strength and/or low physical performance were defined as original sarcopenia.

### Extraction of Radiomic Features

**FIGURE 1:**
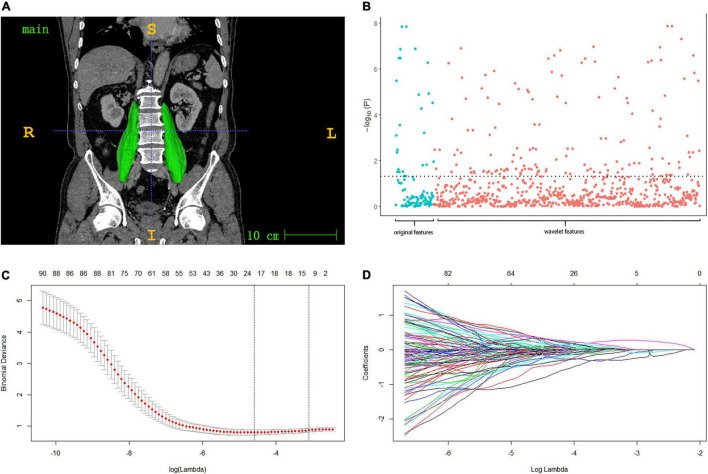
**(A)** Muscle ROI: after the muscle region is labeled in ITK-SNAP software, semi-automatic drawing is adopted to check the psoas major muscle region and 3d reconstruction. **(B)** Manhattan plot presenting the strength of association –log10 (*p*-value) of spearman’s rho between the psoas radiomic features and the original sarcopenia (diagnosed by SMI, grip strength and walking speed). **(C,D)** Model with smallest lambda was selected lambda 0.0451, ln (lambda) –3.098, and 14 features selected from the lasso were included in the subsequent radiomic score model.

All patients underwent enhanced abdominal computed tomography (CT). A 64-slice spiral CT scanner (Siemens; Erlangen, Germany) was used with a slice thickness of 0.75–1.25 mm and covering the entire abdomen (250–400 slices). The portal phase CT image was uploaded to ITK-SNAP (19) (version 3.8.0^1^) for semi-automatic drawing of the psoas major muscle region and 3D reconstruction (Figure 1A). The muscle area was drawn by two experienced researchers and examined by another radiologist. The outline image of the patient’s region of interest (ROI) is shown in Figure 1A. The original CT image and ROI were preserved as medical digital imaging files in NRRD formats, and PyRadiomics 18 was used for automatic feature extraction in the Python environment (version 3.7.2^2^). The detailed list of radiomic muscle feature extraction parameters adjustment and Z-score standardized processing is shown below.

### Parameter Adjustment and Z-Score Standardization

The radiomic feature extraction parameters were adjusted as follows: normalize: false; padDistance: 10; resampledPixelSpacing: [1, 1, 1], Original: []; Wavelet: []. All other parameters used default settings. The PyRadiomics package with extraction parameters mentioned above process the original CT image and ROI image and to produce radiomic features for each patients. Eighteen first-order features, 14 shape-related features (shape-3D), 22 gray level co-occurrence matrix features, 22 gray level run length matrix features, 16 gray level size zone matrix features, 5 neighboring gray tone difference matrix features, and 14 gray level dependence matrix features were extracted, with a total of 833 features after wavelet transformation (20). All features of all patients were standardized by Z-score (with the mean and standard deviation of the training group). The method used is as follows. For the sequence*X* = [*x*_1_,*x*_2_,…,*x*_*n*_], the formula for Z-score transformation was as follows: $y_{i}=\frac{x_{i}-\bar{x}}{\sigma \,\left(X\right)}$, of which$\bar{x}=\frac{1}{n}\,\sum_{i=1}^{n}x_{i}$, and $\sigma \,\left(x\right)=\sqrt{\frac{n}{\left(n-1\right)}\,\sum_{i=1}^{n}{\left(x_{i}-\bar{x}\right)}^{2}}$. Finally, the standard sequence was given by *Y* = [*y*_1_,*y*_2_,…,*y*_*n*_].

### Screening of Valuable Characteristics and Establishment of Diagnostic Model for Radiomic Sarcopenia

In the screening of sarcopenia-related radiomic characteristics and establishment of a diagnostic model, 550 patients were divided into training and validation cohorts chronologically. Eight hundred and thirty-three characteristics in the training group were screened preliminary using Spearman’s test (*p* < 0.05) (21). The remaining features were analyzed by using least absolute shrinkage and selection operator (LASSO) regression analysis. Radiomic scores were calculated using the LASSO regression built by the training cohort. The patients in the validation group were considered for accuracy verification of the radiomic diagnostic model. Based on the extracted radiological features of sarcopenia, the diagnostic criteria for radiomic sarcopenia in both groups were established.

### Statistical Analysis

The Kolmogorov–Smirnov test was carried out to evaluate the distributions of continuous data. Normally distributed data were expressed as means ± standard deviations (SDs). The valuable radiomic features selected by Spearman’s test were used for LASSO regression with the R package “glmnet” (22). The t-test was used to compare continuous data (age and BMI) between the training and validation cohorts, while the chi-squared test or Fisher’s exact test were used to compare categorical data. Multivariate logistic adjusted regression analysis based on the findings of the univariate analysis was performed to validate the independent correlation between radiomic sarcopenia and postoperative complications. Receiver operator characteristic (ROC) curve analysis (23) was used to compare different diagnostic models, and the Kaplan–Meier method (24) was used to estimate the survival curve. Significance was determined by a threshold of *p* < 0.05. Spearman’s test and LASSO regression were performed using R software (version 3.6.0^3^). Logistic analysis, ROC curve analysis, and Kaplan–Meier method were processed through SPSS version 22.0 (IBM Corp, Armonk, NY, United States).

## Results

### Clinical Characteristics of Patients

In this study (Table 1), the training cohort had 261 patients (201 males, 60 females) while the validation cohort had 289 patients (202 males, 87 females). There was no significant difference in sex between the cohorts (*p* = 0.06). The mean age of the training cohort was 64.6 ± 10.2 years and 64.9 ± 10.7 years in the validation cohort. No significant difference in age was found between the two cohorts (*p* = 0.698). There was no significant difference in preoperative nutritional indicators between the two cohorts, such as BMI (*p* = 0.522) or NRS-2002 score (*p* = 0.117). Preoperative assessments of sarcopenia, such as SMI (*p* = 0.561), low grip strength (*p* = 0.208), and low walking speed (*p* = 0.177), were not significantly different between the groups. The number of patients who chose laparoscopic surgery in the validation cohort was higher than that in the training cohort (38.1 vs. 27.6%, *p* = 0.009). There were no significant differences in tumor characteristics or prognosis between the two cohorts, including total gastric resection (*p* = 0.662), anastomotic type (*p* = 0.251), TNM stage (*p* = 0.172), and postoperative complications (*p* = 0.399). The consistency of the basic clinical characteristics between the two groups can make our results in the verification group more reliable.

**TABLE 1:** Clinical characteristics of patients.

Factors	Training group	Validation group	*P-value*
Age, years	64.6 ± 10.2	64.9 ± 10.7	0.698
BMI, kg/m^2^	22.6 ± 3.1	22.8 ± 3.0	0.522
Gender			0.060
Female	60 (23.0%)	87 (30.1%)	
Male	201 (77.0%)	202 (69.9%)	
NRS-2002 score			0.117
1–2	164 (62.8%)	204 (70.6%)	
3–4	78 (29.9%)	72 (24.9%)	
5	19 (7.3%)	13 (4.5%)	
Low SMI			0.561
No	183 (70.1%)	196 (67.8%)	
Yes	78 (29.9%)	93 (32.2%)	
Low grip strength			0.208
No	195 (74.7%)	202 (69.9%)	
Yes	66 (25.3%)	87 (30.1%)	
Low walking speed			0.177
No	222 (85.1%)	257 (88.9%)	
Yes	39 (14.9%)	32 (11.1%)	
Laparoscopic surgery			0.009
No	189 (72.4%)	179 (61.9%)	
Yes	72 (27.6%)	110 (38.1%)	
Total gastric resection			0.662
No	156 (59.8%)	178 (61.6%)	
Yes	105 (40.2%)	111 (38.4%)	
Combined resection			0.263
No	233 (89.3%)	266 (92.0%)	
Yes	28 (10.7%)	23 (8.0%)	
Anastomotic type			0.251
Roux-en-Y	127 (48.7%)	130 (45.0%)	
Billroth I	98 (37.5%)	104 (36.0%)	
Billroth II	36 (13.8%)	55 (19.0%)	
TNM stage			0.172
I	82 (31.4%)	112 (38.8%)	
II	52 (19.9%)	56 (19.4%)	
III	127 (48.7%)	121 (41.9%)	
Postoperative complications			0.399
No	199 (76.2%)	228 (79.2%)	
Yes	62 (23.8%)	61 (20.8%)	

*Data shown in the table: Mean ± SD/N (%).*

*BMI, body mass index; NRS-2002, nutritional risk screening; SMI, skeletal muscle index; TNM, tumor–node–metastasis.*

### Feature Screening and Establishment of a Radiomic Diagnostic Model of Sarcopenia

Initially, 115 of 833 radiomic features associated with original sarcopenia were screened by Spearman’s rho (*p* < 0.05) (Figures 1A,B). LASSO regression was used to further reduce the number of features. At a lambda of 0.0451 (lambda with the minimal binomial deviance plus one standard error), 14 features were included in the final LASSO model (Figures 1C,D). Radiomic scores for each group were calculated by the model built with the training cohort. The cut-off value of the radiomic score was selected by the maximum Youden’s index (sensitivity + specificity - 1) (25) for predicting postoperative complications in the training cohort, which was set to –1.59.

### Correlation Between Radiomic Sarcopenia and Original Sarcopenia

**FIGURE 2:**
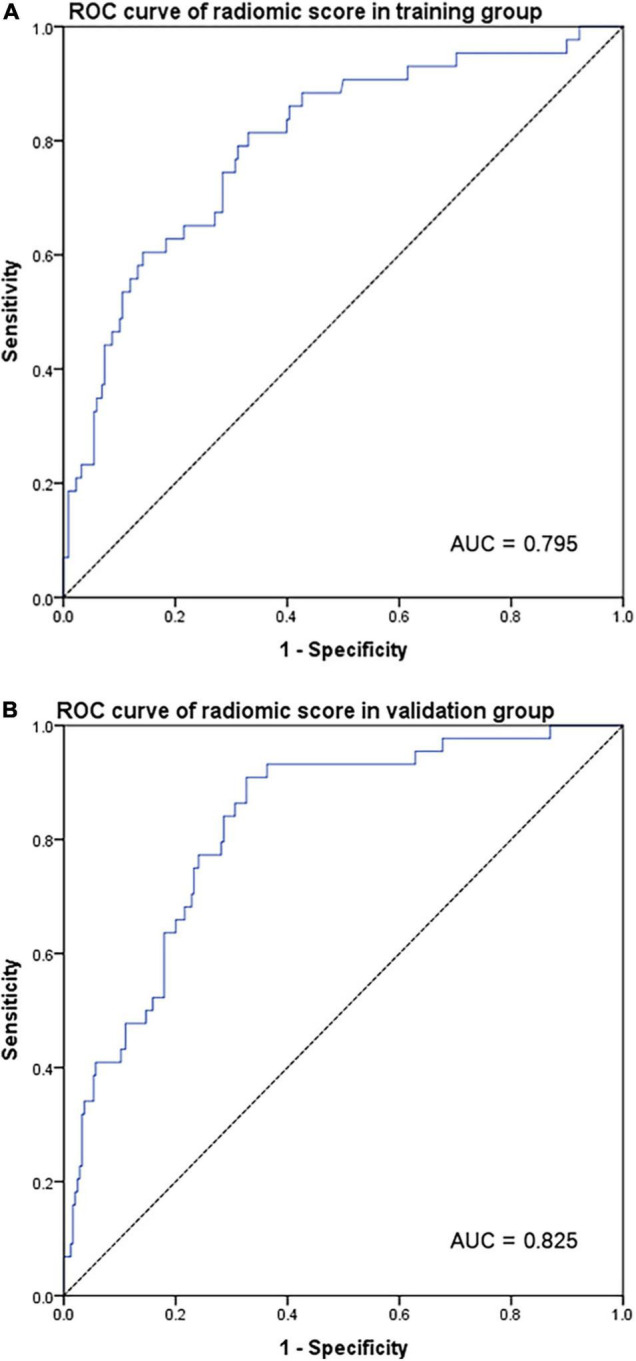
ROC curve for radiomic score according to original sarcopenia in the training group **(A)** and validation group **(B)**.

In the training group (Figure 2A), using original sarcopenia as a reference value, the area under the ROC curve (AUC) for the radiomic score was 0.795 (95% CI = 0.720–0.870, *p* < 0.001). The validation cohort (Figure 2B) also had a high AUC of 0.825 (95% CI = 0.763–0.887, *p* < 0.001). Radiomic scores were both correlated with original sarcopenia in both the training and validation cohorts. Supplementary Figures 2A–C show the correlations between radiomic scores and low grip strength, low walking speed, and low SMI in the validation cohort, respectively. Supplementary Figures 2D–F show similar correlations in the training cohort.

### Univariate and Multivariate Analysis of Short-Term Complications

As shown in Table 2, in the training cohort, univariate analysis showed that radiomic sarcopenia [odds ratio (OR), 3.3; 95% CI 1.8–6.0; *p* < 0.001], age (OR, 2.3; 95% CI, 1.3–4.1; *p* = 0.006), endoscopic surgery (OR, 0.8; 95% CI, 0.4–1.4; *p* = 0.018), combined resection (OR, 2.7; 95% CI, 1.2–6.2; *p* = 0.015), and Billroth I (OR, 0.5; 95% CI, 0.2–0.9; *p* = 0.025) were significantly correlated with grade II and above complications. There was no significant correlation between the occurrence of postoperative complications and sex, BMI, NRS-2002, total gastric resection, or TNM stage. In the multivariate analysis after adjustment for potential related factors, endoscopic surgery (*p* = 0.032) and radiomic sarcopenia (*p* = 0.003) remained independent predictors of major postoperative complications.

**TABLE 2:** Univariate and multivariate analysis of postoperative complications.

Factors	Training group			Validation group		
	Univariate analysis		Multi- analysis *c* value	Univariate analysis		Multi- analysis *P-value*
	OR (95%CI)	*P-value*		OR (95%CI)	*P-value*	
Radiomic sarcopenia	3.3 (1.8, 6.0)	<0.001	0.003*	3.6 (2.0, 6.5)	<0.001	<0.001*
Gender		0.197			0.767	
Female	Ref			Ref		
Male	0.7 (0.3, 1.2)	0.197		0.9 (0.5, 1.7)	0.767	
Age > 65 years	2.3 (1.3, 4.1)	0.006	0.142	1.9 (1.0, 3.4)	0.037	0.135
BMI < 18.5 kg/m^2^	1.8 (0.7, 4.5)	0.198		2.7 (1.1, 6.6)	0.028	0.622
NRS-2002 score		0.106			<0.001	0.013*
1–2	Ref			Ref		
3–4	1.3 (0.7, 2.5)	0.39		2.6 (1.4, 4.9)	0.026	0.005
5	2.8 (1.0, 7.5)	0.04		4.8 (1.5, 15.2)	0.079	0.112
Laparoscopic surgery	0.8 (0.4, 1.4)	0.018	0.032*	0.8 (0.4, 1.4)	0.397	
Total gastric resection	1.6 (0.9, 2.8)	0.135		1.8 (1.0, 3.2)	0.040	0.762
Combined resection	2.7 (1.2, 6.2)	0.015	0.124	6.1 (2.5, 14.6)	< 0.001	<0.001*
Anastomotic type		0.007	0.177		0.013	0.028*
Roux-en-Y	Ref			Ref		
Billroth I	0.5 (0.2, 0.9)	0.025	0.154	0.6 (0.3, 1.1)	0.093	0.316
Billroth II	1.7 (0.8, 3.8)	0.162	0.450	0.3 (0.1, 0.7)	0.008	0.010
TNM stage		0.173			0.007	0.050*
I	Ref			Ref		
II	1.5 (0.6, 3.5)	0.393		3.3 (1.5, 7.5)	0.004	0.015
III	1.9 (1.0, 3.8)	0.065		2.5 (1.2, 5.1)	0.011	0.182

*BMI, body mass index; NRS-2002, nutritional risk screening; TNM, tumor–node–metastasis; OR, odds ratio; CI, confidence interval; ref, reference.*

*Statistically significant in multivariate analysis.

In the validation cohort, univariate analysis showed that radiomic sarcopenia (OR, 3.6; 95% CI, 2.0–6.5; *p* < 0.001), age (OR, 1.9; 95% CI, 1.0–3.4; *p* = 0.037), BMI (OR, 2.7; 95% CI, 1.1–6.6; *p* = 0.028), NRS-2002 (OR, 2.6; 95% CI, 1.4–4.9; *p* = 0.028), total gastric resection (OR, 1.8; 95% CI, 1.0–3.2; *p* = 0.04), combined resection (OR, 6.1; 95% CI, 2.5–14.6; *p* < 0.001), anastomotic type (*p* = 0.013), and TNM stage (*p* = 0.007) were associated with short-term prognosis. The multivariate analysis showed that radiomic sarcopenia (*p* < 0.001), NRS-2002 (*p* = 0.013), combined resection (*p* < 0.001), anastomotic type (*p* = 0.028), and TNM stage (*p* = 0.050) were independent predictive risk factors for major complications after surgery.

### Differences in Predicting Tumor Prognosis Between the Two Sarcopenia Evaluation Methods

**FIGURE 3:**
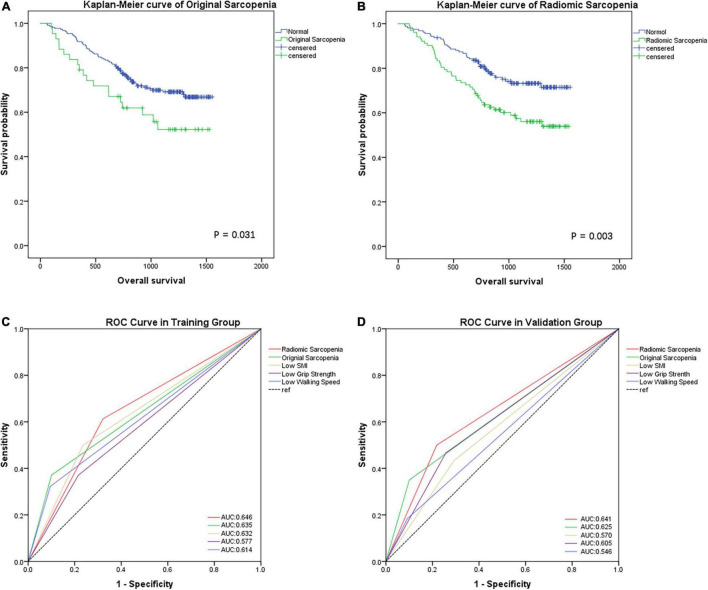
Kaplan–Meier analysis for overall survival (days) of GC patients in the training group according to original sarcopenia **(A)** and radiomic sarcopenia **(B)**. ROC curves for the preoperative characteristics of different sarcopenia models according to short-term complications after gastrectomy in training group **(C)** and validation group **(D)**.

In the training group, based on Kaplan–Meier curve analysis, patients with original sarcopenia had a worse overall survival (OS) than normal patients [hazard ratio (HR), 1.741; 95% CI, 1.044–2.903; *p* = 0.031) (Figure 3A). Meanwhile, radiomic sarcopenia had a better prognostic value: the OS time of those with radiomic sarcopenia was significantly shorter than normal patients (HR, 1.880; 95% CI, 1.225–2.885; *p* = 0.003) (Figure 3B).

SMI, grip strength, and walking speed were correlated with postoperative complications in both cohorts (AUCs: 0.632, 0.577, and 0.614, respectively in the training cohort; 0.570, 0.605, 0.546, respectively, in the validation cohort). Moreover, original sarcopenia was more accurate than other indicators (Figures 3C,D). However, radiomic sarcopenia had a higher AUC in predicting short-term complications than original sarcopenia in both cohorts (AUCs: 0.646 vs. 0.635 in the training cohort; 0.641 vs. 0.625 in validation cohort) (Figures 3C,D).

## Discussion

The incidence of sarcopenia is 5–13% in people aged 60–70 years and may reach 50% in people aged >80 years (26). In China, the prevalence among men aged ≥70 years is approximately 12.3%, while the prevalence among women is approximately 7.6% (27). In our previous study, we observed a poor clinical prognosis in patients with sarcopenia, and sarcopenia was an independent predictor of postoperative complications and poor long-term survival (28, 29).

However, original sarcopenia definition does not only needs to consider muscle mass but also muscle strength and physical performance. Pre-diagnosis of sarcopenia requires image reading and specialized personnel, which involves time and energy in analyzing grip strength and walking pace. CT is a useful muscle mass analysis tool for diagnosing traditional sarcopenia; however, the muscle status on CT has not been well analyzed. In this study, we proposed the concept of radiomic sarcopenia through features of sarcopenia extracted from CT. This new type of sarcopenia index can be extracted using a three-dimensional imaging method, which objectively examines muscle mass, muscle strength, and physical performance. Compared with original sarcopenia, radiomic sarcopenia is based on the extraction of image histology features with objectivity and rigor, which can reduce the diagnostic deviation caused by subjective evaluation of grip strength and walking pace. Simultaneously, it has a higher sensitivity and specificity in predicting postoperative complications and is also an independent predictor of major postoperative complications and poor long-term survival.

Radiomic technology captures tissue heterogeneity in a non-invasive manner and uses automated high-throughput data feature extraction algorithms to convert image data into high-resolution, minable image feature data (30, 31). In sarcopenia assessment, after measuring the muscle mass from CT, we also need to evaluate muscle strength and physical performance, which require more time and energy. Meanwhile, radiomic sarcopenia assessment requires a shorter time, given that the assessment results can be obtained instantaneously after CT measurements. Radiomic sarcopenia does not require clinicians to measure a patient’s grip strength or usual gait speed, thereby reducing manpower and medical resources. Clinically, the use of radiomics in diagnosing sarcopenia can help clinicians identify the presence of sarcopenia more quickly and conveniently. Clinicians can implement nutritional and clinical interventions based on this new diagnostic method to establish a better perioperative diagnosis and treatment system for GC patients.

In this study, both in the training group and the validation group, radiomic sarcopenia had a high AUC for predicting complications. Although there was no obvious improvement in sensitivity and specificity compared with original sarcopenia, radiomic sarcopenia had a similar accuracy and were faster to obtain and more convenient to use. In Table 1, although the number of patients in the cohorts was different, there was no obvious difference in basic characteristics, which can indicate that our results in the validation group are credible. In terms of long-term survival, owing to the lack of survival follow-up time for GC patients from 2017 to 2019 (the validation cohort), the survival analysis was only performed for the training cohort and not the validation cohort.

To the best of our knowledge, this study is the first to propose the use of radiomic sarcopenia in predicting the short-term and survival prognosis of GC patients after surgery. However, this study had some limitations. Owing to limited resources, we included only 550 patients, this sample size may not be sufficiently representative. Therefore, in future research, we need to include more patients and more centers for comprehensive and systematic verification. We look forward to the establishment of an index-like radiomic diagnosis of sarcopenia through image feature extraction, instead of a subjective scale, to objectively evaluate the nutritional status of the body, this will help in reducing the diagnosis cost and ensure quicker and more accurate diagnosis.

## Conclusion

Extracted sarcopenia features based on CT can predict the long-term survival and short-term complications of GC patients after surgery. The accuracy of our model was verified using training and verification cohorts. Compared with original sarcopenia, radiomic sarcopenia can not only effectively improve the accuracy of survival and complication prediction, but also shorten the time and steps of traditional screening, reducing the subjectivity of sarcopenia assessment.

## Data Availability Statement

The raw data supporting the conclusions of this article will be made available by the authors, without undue reservation.

## Ethics Statement

The studies involving human participants were reviewed and approved by the Ethics Committee of the two affiliated hospitals of Wenzhou Medical University. Written informed consent for participation was not required for this study in accordance with the national legislation and the institutional requirements.

## Author Contributions

XS and W-JC contributed to conception and design of the study. L-BX, Z-XH, and H-HZ contributed to the acquisition of the data. X-DC, W-TZ, M-MS, and Y-QC contributed to the analysis and interpretation of the data. X-DC, W-TZ, and L-BX drafted the manuscript. Z-SL revised the manuscript. All authors critically revised the manuscript and gave final approval of the version to be submitted.

## Conflict of Interest

The authors declare that the research was conducted in the absence of any commercial or financial relationships that could be construed as a potential conflict of interest.

## Publisher’s Note

All claims expressed in this article are solely those of the authors and do not necessarily represent those of their affiliated organizations, or those of the publisher, the editors and the reviewers. Any product that may be evaluated in this article, or claim that may be made by its manufacturer, is not guaranteed or endorsed by the publisher.

## Footnotes

1
http://www.itksnap.org/

2
https://python.org/

3
http://www.R-project.org

## Supplementary Material

Supplementary Figure 1 The flowchart of participant.

Supplementary Figure 2 ROC curve for radiomic score according to low grip strength, low walking speed, and low SMI in the training group **(A–C)** and validation group **(D–F)**.
