# Supplementary figures and images for: Establish a New Diagnosis of Sarcopenia Based on Extracted Radiomic Features to Predict Prognosis of Patients With Gastric Cancer
Source: Front Nutr. 2022 Jun 28;9:850929. doi: 10.3389/fnut.2022.850929 (PMC9276522; doi:10.3389/fnut.2022.850929)

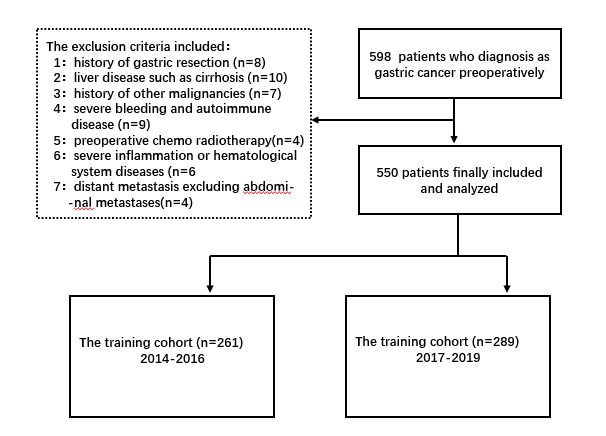

Supplement: Supplementary Figure 1 — The flowchart of participant. [file Image_1.PNG]

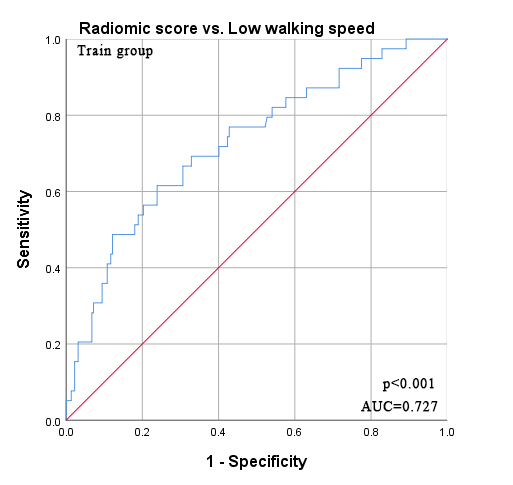

Supplement: Supplementary Figure 2 — ROC curve for radiomic score according to low grip strength, low walking speed, and low SMI in the training group (A–C) and validation group (D–F). [file Image_2.JPEG]

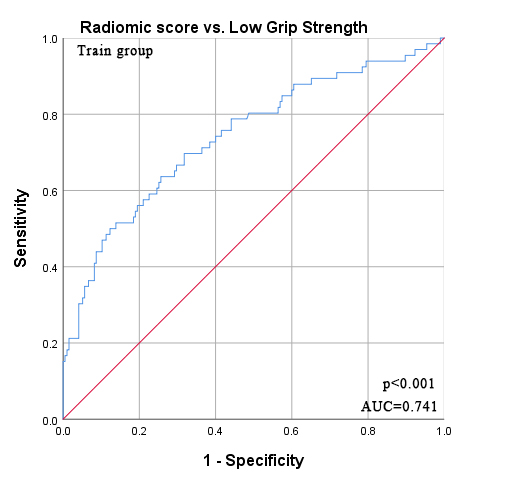

Supplement: Supplementary file 3 [file Image_3.JPEG]

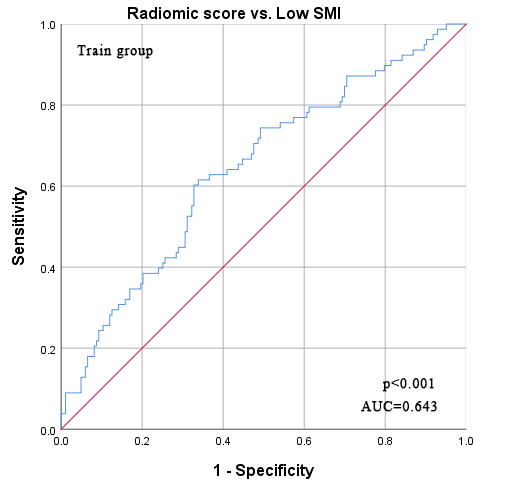

Supplement: Supplementary file 4 [file Image_4.JPEG]

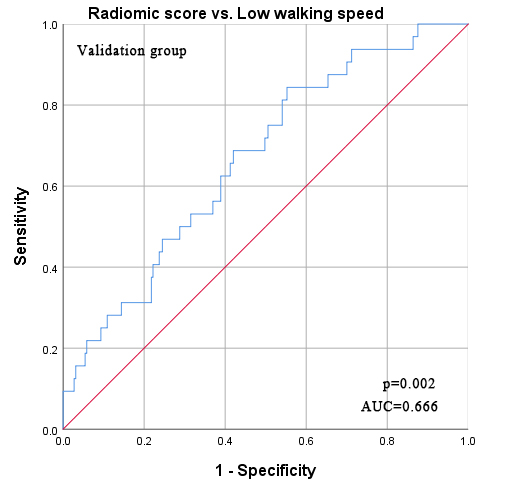

Supplement: Supplementary file 5 [file Image_5.JPEG]

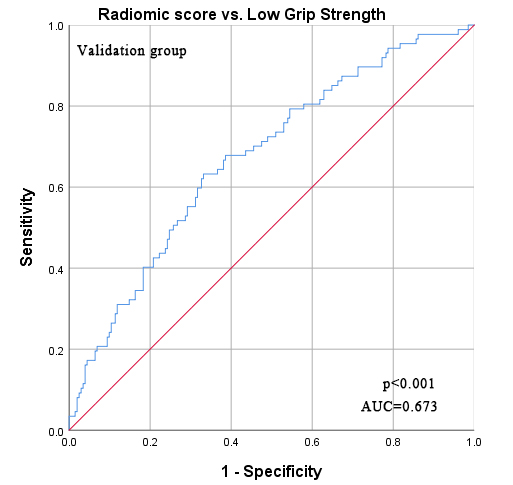

Supplement: Supplementary file 6 [file Image_6.JPEG]

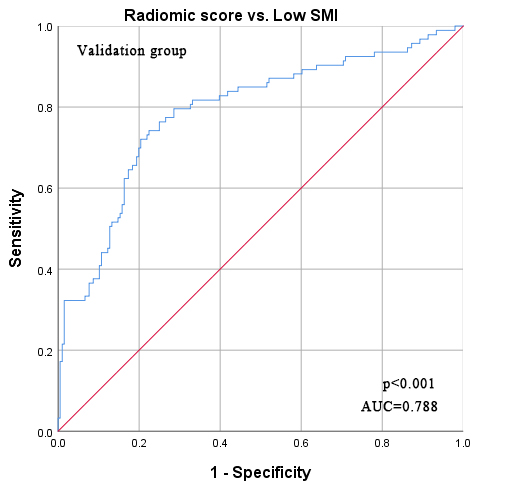

Supplement: Supplementary file 7 [file Image_7.JPEG]
